# Clinical Spectrum, Diagnosis, and Treatment Outcome in Individuals With Intravascular Large B-cell Lymphoma Affecting the Nervous System: A Case Series

**DOI:** 10.7759/cureus.37007

**Published:** 2023-04-01

**Authors:** Ning Zhong

**Affiliations:** 1 Neurology, Kaiser Permanente Sacramento Medical Center, Sacramento, USA; 2 Neurology, University of Washington Medical Center, Seattle, USA

**Keywords:** cerebro-vascular accident (stroke), myopathy, central nervous system, rituximab, intravascular large b-cell lymphoma

## Abstract

We treated five patients, three females, and two males, with intravascular lymphoma that affected the central or peripheral nervous systems. We reviewed their clinical, laboratory, neuro-imaging, and pathological data and treatment outcomes. The median age of onset was 60 years, with a range of 39 to 69 years. Three patients presented with central nervous system symptoms only, such as confusion, aphasia, seizure, stroke, and ataxia. Three patients presented with systemic lymphoma stage B symptoms, one with peripheral nervous system symptoms, and one with multi-organ failure. Brain imaging revealed white matter lesions, infarcts, hemorrhages, or combinations. Histology showed CD20-positive B-lymphocytes confined to small-size vessels in autopsy or biopsy specimens from the brain or muscle, confirming the diagnosis of intravascular large B-cell lymphoma (IVLBL). The patient with multi-organ failure had diffuse infiltration to the spleen, liver, and kidney. Three patients died within three to four months after the clinical presentation and were diagnosed at autopsy. The other two were diagnosed by biopsy and underwent chemotherapy CHOP-R (cyclophosphamide, hydroxydaunorubicin, Oncovin, and prednisone) or MTX (methotrexate)+Rituximab. The median survival of the chemotherapy patients was 17.5 months, compared to three to four months in those who did not receive chemotherapy. Although IVLBL has distinct pathological features, its clinical presentation can be variable. The patient's best chance for survival depends on the early pathological diagnosis and prompt, aggressive chemotherapy.

## Introduction

Intravascular large B-cell lymphoma (IVLBL) is defined as the proliferation of monoclonal B lymphocytes within the lumina of small vessels and capillaries [1]. The current WHO classification of hematopoietic diseases has recently classified IVLBL as a distinct, rare subtype of extranodal diffuse B-cell/non-Hodgkin's lymphoma [2]. Its estimated incidence is less than one per million based on its appearance in the literature, and it occurs equally in women and men. The median age of onset is between 60 and 70 years [3]. The disease was first reported by Pfleger and Toppeiner in 1959 and it was described as “angioendotheliomatosis proliferaus systemisata” [4]. The lymphoid nature of this entity was confirmed by Wick et al. in 1986 [5].

Since its first publication nearly 60 years ago, the vast majority of IVLBL literature consisted of single or small series case reports. However, a few groups have reported fairly large series, revealing IVLBL to be extremely heterogeneous in its clinical presentation [6-12]. It is well recognized that IVLBL is a fatal disease characterized by an aggressive clinical course and poor prognosis. Two clinical variants have been proposed, 1) European variant cases diagnosed in Western countries displayed central nervous system and cutaneous involvement [7] and 2) cases diagnosed from Asian countries showed hemophagocytic syndrome, bone marrow involvement, and multiorgan infiltration [11].

Clinical symptoms of the disease are related to the specific organ involved [13]. According to Autopsy data, IVLBL pathologically involves the nervous system in 60%-85% of the patients [14] and over one-third of patients present with neurological symptoms as the first manifestation [1,14]. A broad spectrum of neurological symptoms has been reported, including mental status changes, seizures, cerebral vascular events, myelopathy, or peripheral neuropathy/myopathy [1,2,14]. Progressive deterioration, mimicking an inflammatory, vascular, or degenerative process, is often common. The clinical course is usually fatal in a few weeks to months if left untreated, and the clinical variant and presentation do not correlate with prognosis or treatment outcome [15]. The patient’s optimal chance for survival relies upon early pathological diagnosis and prompt, aggressive chemotherapy [16,17]. We presented a case series of IVLBL with different clinicopathological manifestations and treatment outcomes.

## Case presentation

Patients and methods

Five patients were included in the study, with two patients diagnosed from tissue biopsy and the other three diagnosed by autopsy at postmortem examination, based on the World Health Organization (WHO) classification. Demographic, clinical, imaging, and pathological data were reviewed, and overall survival was calculated from the first clinical sign to death or the last follow-up date. Two patients who received chemotherapy were followed up for six months and 15 months after treatment, respectively. This case series report has been approved by the University of Washington Human Subject Division and adheres to the provisions of the Declaration of Helsinki.

Case report

Case 1

A 39-year-old female presented with a persistent headache and fever three months before admission. She received treatment for sinusitis but did not show any improvement. Following a comprehensive workup, she was diagnosed with systemic lupus erythematosus based on positive antinuclear antibodies (ANAs) with a speckled pattern. She responded briefly to a trial of daily prednisone 60mg. However, one day before admission, the patient's symptoms returned with fever, malaise, headache, and confusion. She was found to have severe anemia, thrombocytopenia, and renal insufficiency, with a creatinine level of 2.1 and elevated LDH > 9,000. Tentative diagnosis included thrombotic thrombocytopenic purpura and lupus cerebritis. She received a three-day course of plasma exchange and pulsed methylprednisolone, but there was no significant clinical improvement. During her 10-day hospital stay, she continued to experience intermittent severe headaches and mental status fluctuations, including confusion, disorientation, and combative behavior. A brain MRI revealed an abnormal T2/FLAIR signal in the superior vermis and an enlarged non-enhancing pituitary gland.

Due to the patient's severe anemia, a peripheral blood smear and bone marrow biopsy were performed, but neither test revealed any sign of malignancy. A CT scan of her body showed abdominal organomegaly (enlarged liver, spleen, and kidney). As part of further diagnostic testing, she underwent a kidney and abdominal lymph node biopsy. Unfortunately, following the procedure, the patient developed DIC, peritoneal bleeding, and multi-organ failure, which led to her passing away three days later.

The gross examination of the brain revealed several abnormalities. Firstly, the pituitary gland was mildly enlarged, and the cut surfaces had a distinct yellow discoloration. Secondly, parasagittal sections of the left cerebellum and horizontal sections of the right cerebellum and brainstem showed a hemorrhagic, necrotic area in the superior vermis, with shrunken folia in the approximate distribution of the superior cerebellar artery. At the apex of this infarct in the white matter below the folia, there was a 0.2 cm, distinctly circular, red infarct. Further examination of brain sections revealed multi-vessel involvement with lymphoma cells. The pituitary gland also had a large area of coagulative necrosis consistent with an infarct, and the pituitary vasculature contained lymphoma cells associated with the clot. Moreover, sections taken from the vermis infarct showed a vessel containing a clot that appeared to be organizing with multiple endothelialized lumina, and there were lymphoma cells present within the recanalized lumen. The autopsy revealed disease involvement in every major organ system, along with extensive intravascular involvement.

Case 2

A 61-year-old male patient presented with intermittent fever, fatigue, a generalized weakness for six months, and a 15-lb weight loss. During his illness, he also developed progressive bilateral lower extremity peripheral edema and numbness/tingling. A thorough evaluation revealed microcytic anemia, ascites, and pleural effusion. Bone marrow biopsy revealed a population of small B-cells, which were kappa-restricted with low CD19, intermediate CD20, and bright CD45 expression, but no sign of malignancy. He was treated with an unknown course of oral prednisone, which did not produce any obvious effect. Despite the treatment, the patient continued to experience intermittent fever, and fatigue and developed bilateral foot drops. Three days before admission, he experienced a mental status change with increased somnolence and confusion. Lab tests showed only signs of anemia, elevated ESR (erythrocyte sedimentation rate), mildly elevated LDH (lactate dehydrogenase), and a slightly positive ANA. MRI imaging did not reveal any acute intracranial changes.

Due to low extremity sensory deficits and muscle atrophy/weakness, the patient underwent an EMG (electromyography), which revealed severe inflammatory myopathy. To further investigate, the right sural nerve and right gastrocnemius biopsies were performed. Results showed a striking infiltration of lymphoma cells within the lumina of intramuscular blood vessels, indicating IVLBL. The immunocytochemical analysis confirmed the presence of CD20, CD79a, and CD5 positive atypical large B-cell population in clumps within the lumina of intramuscular blood vessels. The muscle biopsies also revealed many myofibers with atrophy and others with compensatory hypertrophy, but no evidence of denervation or reinnervation, suggesting the absence of neuropathy.

The patient commenced the cyclophosphamide, hydroxydaunorubicin, Oncovin, and prednisone (CHOP) chemotherapy regimen without vincristine and experienced substantial improvement in symptoms, eventually regaining baseline functional status. Four months after diagnosis and initiation of chemotherapy, relapsing symptoms of fatigue and generalized weakness manifested. The patient subsequently received two courses of MIME and three doses of rituximab, resulting in an improvement in symptoms and functional status. During the last follow-up approximately one year after the initial presentation, the patient remained free of tumor recurrence.

Case 3

A 69-year-old female patient with a history of recurrent focal weakness, neglect, and aphasia presented with 10 days of lethargy, intermittent fever, ataxia, and confusion. On the day of admission, the patient became increasingly confused, and restless, and developed right-sided paresis. An MRI revealed an acute left thalamic infarct. Lab tests revealed anemia, an elevated ESR and LDH, and CSF showed mild pleocytosis and an elevated protein level. Despite treatment, the patient remained encephalopathic out of proportion to her lacunar infarct. Two weeks later, the patient's mental status acutely deteriorated, with new gaze deviation. A repeat MRI revealed multiple bilateral acute infarcts. A cerebral angiogram ruled out vasculitis. MRI spectroscopy showed no evidence of mitochondrial disease (no abnormal accumulation of lactate in the brain parenchyma). The patient was treated with IV methylprednisolone and IV infliximab, resulting in transient improvement of mental status, but followed by rapid deterioration.

The patient was deceased three months after the initial presentation. An autopsy and pathology exam revealed the presence of CNS IVLBL.

Case 4

**Figure 1:**
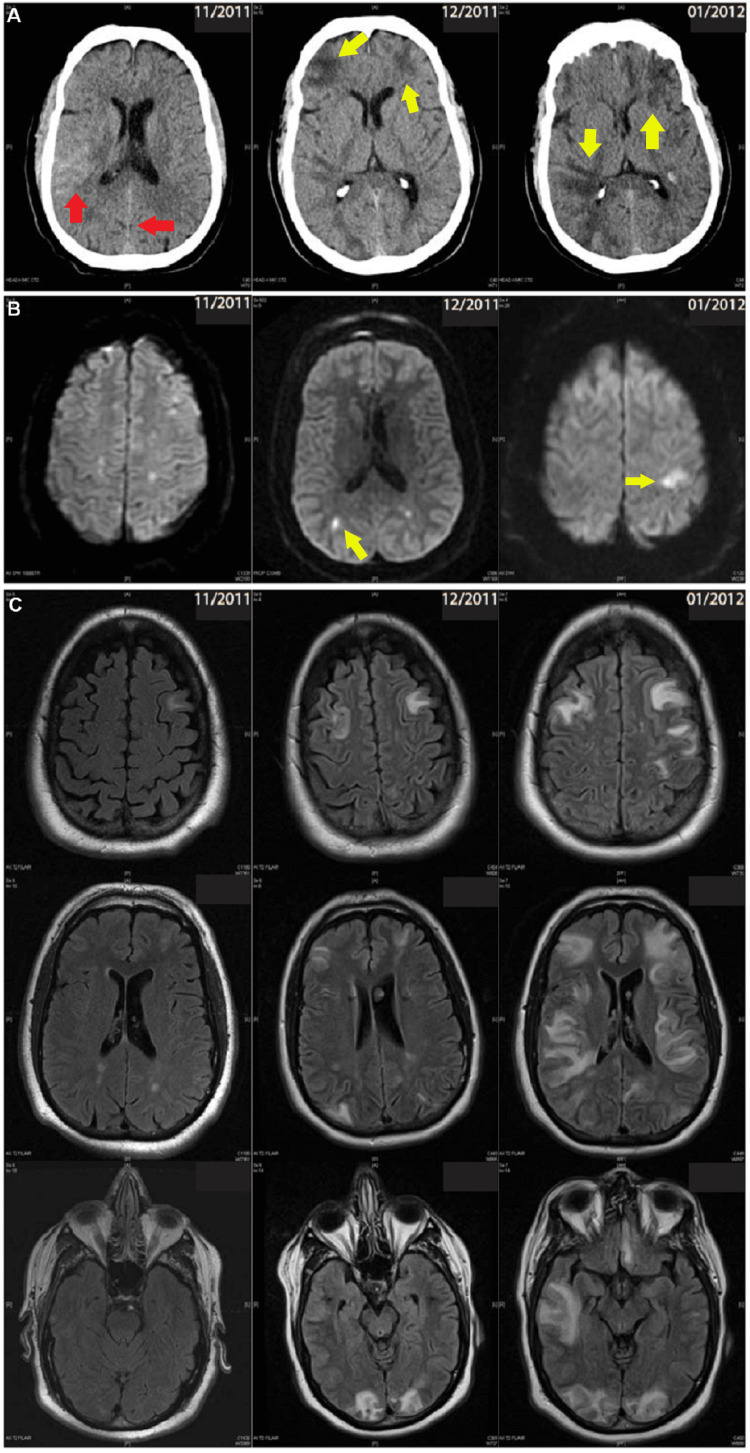
Dynamic progression of different types of brain lesions in a patient who did not receive chemotherapy (case 4). (A) SAH (red arrow), IPH (red arrow), and cerebral edema (yellow arrow) in the patient with a different clinical stage, (B) multi-foci of restrict diffusion representing acute ischemic infarct (light green arrow), (C) progression of T2/FLAIR hyperintensity lesions in a different clinical stage. SAH - Subarachnoid Hemorrhage; IPH - Intraparenchymal Hemorrhage

Three months prior to admission, a 60-year-old male patient presented with a severe headache and a new-onset seizure. CT head revealed bilateral subarachnoid hemorrhage (SAH, more prominently seen in the right frontal lobe and bilateral occipital lobe, Figure 1A). Brain MRI showed a watershed infarct (Figure 1B). Two months before admission, he began experiencing fatigue, difficulty with word-finding, recurrent seizures, weakness, and a right-hand tremor. A repeat brain MRI showed new foci of cerebral infarcts involving the bilateral frontal, temporal, parietal, and occipital lobes (Figure 1B), as well as small areas of acute hemorrhage in the posterior occipital lobes seen in the head CT (Figure 1A). Cerebral angiography suggested cerebral vasoconstrictive syndrome.

The patient was transferred to our medical center due to worsening confusion, new onset of dysphagia, aphasia, and right facial twitching. Lab tests revealed elevated ESR and LDH. CSF analysis showed normal cell differentiation and flow cytometry did not detect malignancy. A non-contrast head CT revealed a new left temporoparietal intraparenchymal hemorrhage. Serial brain MRIs showed progressive and new acute restricted diffusion, as well as diffuse patchy T2/FLAIR hyperintensities in subcortical locations with enhancement (Figure 1C). The patient received treatment with IV methylprednisolone for five days, and his mental status and aphasia slightly improved for a brief period. However, his mental status continued to deteriorate, and he became comatose. Brain biopsy results were not available until after the patient's death. Five days later, the patient passed away due to diffuse cerebral edema and transtentorial herniation, 3.5 months after the initial presentation.

The brain tissue biopsy revealed multifocal subtotal necrosis with small patches of incomplete neuronal loss and reactive astrocytes. Clusters of mildly atypical lymphoid cells were present within intravascular lumens, although there was no evidence of vasculitis. Immunohistochemistry staining confirmed the identity of the intravascular cells as CD20+ B-type lymphocytes. A gross examination of the autopsied brain revealed uncal herniation. Patchy dusky discoloration and scattered parenchymal hemorrhages were present in bilateral cerebral hemispheres, brain stem, and cerebellum. Multiple vessels ranging in size from meningeal muscular arteries to capillaries containing lymphoma cells were present in multiple regions of gray and white matter. Immunohistochemistry staining confirmed the identity of the intravascular lymphoma cells as CD20+ cells.

Case 5

A 68-year-old female presented with a six-month history of progressive weakness, confusion, ataxia, and dysgraphia. She also developed a seizure, behavioral arrest, and decreased verbal output. One week prior to admission, the patient acutely deteriorated with difficulty walking, the tendency to fall toward the right, word-finding problems, and difficulty in executive function, short-term memory, and attention. Brain MRI showed contrast enhancement in the cerebellum, with the right side being more prominent than the left, as well as in bilateral frontal lobes. The patient underwent a cerebral angiogram to rule out CNS vasculitis, which was negative. CSF analysis did not show evidence of ongoing inflammation or infection, and flow cytometry and cytology were unremarkable. A brain biopsy was promptly obtained, which showed evidence of intravascular lymphoma. The pathology revealed CD20+ positive B-type lymphocytes within the small vessels and capillaries throughout the biopsied tissue sample. 

After the diagnosis, the patient underwent several diagnostic tests, including bone marrow and skin biopsies, a whole-body CT scan, and an MRI of the spine, which showed no evidence of involvement in other systems. The patient was then treated with eight cycles of induction chemotherapy, consisting of methotrexate and rituximab, followed by 12 cycles of maintenance methotrexate treatment. Within the first three cycles of induction therapy, the patient experienced a remarkable improvement in symptoms, with significant recovery of cognitive function and regained language ability and no further seizure episodes. Although the patient continued to experience mild gait disturbance and headache, repeat MRI showed complete resolution of patchy enhancing lesions in the cortical/subcortical cerebral and cerebellum. The patient has survived for 23 months since the last follow-up, since the initial presentation. Serial MRI brain imaging showed stable post-therapy changes and no signs of recurrent lesions.

Summary of the clinical features

Table 1 and supplementary data summarize the clinical manifestations and relevant diagnostic work-up data of five patients, consisting of three females and two males. The median age of onset was 60 years (range 39-69). All patients exhibited symptoms related to the nervous system, including acute/subacute mental status changes, progressive neurocognitive deficits, language difficulties, focal motor/sensory deficits, ataxia, and seizures. Three patients presented with initial clinical manifestations confined to the CNS. Among the five patients, three also displayed systemic B-symptoms, such as fever, lethargy, and fatigue. One patient suffered from multi-organ failure, while another was found to have severe inflammatory myopathy in the lower extremities, as determined by an electromyography (EMG) study (Table 1).

**Table 1:** Clinical data ESR- erythrocyte sedimentation rate; LDH - lactate dehydrogenase; ANA - antinuclear antibodies; SAH - subarachnoid hemorrhage; CHOP - cyclophosphamide, hydroxydaunorubicin, Oncovin, and prednisone; MIME - methyl-GAG, ifosfamide, methotrexate, etoposide;

Age/Gender	Chief Complaints	Neurological Symptoms	Other signs/symptoms	Lab tests	CSF studies	Neuroimaging	Pathologic Diagnosis	Chemotherapy	Outcome
39/F	3 months of headache, fever, anemia	confusion, disorientation, combative behavior, and severe headache	Recurrent fever, thrombocytopenia, renal insufficiency	Anemia, thrombocytopenia, elevated LDH, ESR; ANA, Anti-dsDNA, SSA, SSB, anti-cardiolipin, glycoprotein, and lupus inhibitor y; bone marrow biopsy showed hypercellularity without malignancy	no pleocytosis	Abnormal T2/FLAIR signal in the superior vermis; enlarged pituitary gland	Pituitary and cerebellar vasculature contains lymphoma cells associated with clots. Autopsy of the body showed extensive disease with involvement of every major organ system and extensive intravascular involvement	Trial of steroids, mental status transiently improved	Died 3.5 months after initial presentation
61/M	6 months of fever, fatigue, extremity weakness, and peripheral edema	Intermittent confusion and increased somnolence, tingling, and numbness in bilateral lower extremities, bilateral foot drop. EMG showed severe inflammatory myopathy	Low-grade fever, weight loss, fatigue, anemia and ascites, pleural effusion	Anemia, ANA, hypoalbuminemia, elevated ESR, and bone marrow biopsies showed a small clonal population of B cells	n/a	Mild periventricular white matter ischemic changes of aging. No sign of acute infarction or enhancing lesions. No intracranial hemorrhage	The muscle showed a striking infiltration of lymphoma cells within the lumina of intramuscular blood vessels. Immunocytochemical identification of CD20, CD79a, CD5 positive atypical large B-cell population in clumps	CHOP with the exclusion of vincristine × 6 cycles, then MIME therapy × 2 cycles during a relapse; also received 3 doses of rituximab	Significant improvement of fatigue, weakness, B-symptoms, and overall functional status with CHOP therapy. Relapsed one year after the initial presentation and was treated with MIME and rituximab, which improved his symptoms and functional status
69/F	10 days of lethargy, intermittent fever, ataxia, and confusion	Headache, vision changes, progressive confusion, right arm drift, and right-sided weakness; deteriorated to obtundation, and gaze deviation History of transient focal weakness, neglect, and expressive aphasia 3 months prior to the admission	Fever, anemia, thrombocytopenia	Anemia, thrombocytopenia, hypoalbuminemia, elevated ESR, LDH; ANA, Anti-dsDNA, SSA, SSB, anti-cardiolipin, glycoprotein, and lupus inhibitory negative	Pleocytosis with elevated protein	Dynamic change with recurrent multiple foci restricted diffusion in the bilateral frontal and parietal lobes, and thalamus, confluent white matter hyper-intensities involving multiple cortical and subcortical locations	The autopsy revealed CD20-positive atypical B-cell within the lumina of the vessel in brain tissue	Trial of steroids, symptoms of mental status transiently improved	Died within 3 months after initial presentation
60/M	2 months of new-onset seizure and progressive confusion	Initially had a severe headache, seizure, bilateral SAH, watershed infarcts; then aphasia, recurrent seizure, weakness/tremor of R hand, new foci of cerebral infarct; progressive confusion and R facial twitching, new left temporoparietal IPH	No apparent systemic symptoms	Elevated ESR, LDH	no pleocytosis; flow cytometry did not show evidence of malignancy	Dynamic changes with recurrent multiple-foci restricted diffusion involving multiple watershed distributions. Diffuse patchy T2/FLAIR signals involving both white matter and cortical areas, with some enhancement. Different stages of SAH and IPH	Both brain biopsy and autopsy confirmed the existence of lymphoma cells within the lumina of blood vessels, and cell identity was confirmed as CD20+ B-type lymphocyte by immunohistochemistry	Trial of steroid treatment, the patient’s mental status and language ability briefly improved	Died within 3.5 months of initial clinical presentation
68/F	6 months of fatigue, confusion, ataxia, and seizure	seizure, presented as blank staring, behavioral arrest, decreased verbal output; then developed ataxia/gait disturbance for two months; symptoms progressively worsening when admitted in July	No apparent systemic symptoms	Elevated LDH; bone marrow biopsy did not show evidence of malignancy	no pleocytosis with slightly elevated protein	Patchy cortical and subcortical areas of T2/FLAIR signal abnormality as well as the corresponding patchy enhancement within the frontal lobe and cerebellum	Pathology on brain biopsy showed evidence of intravascular lymphoma, CD20+ positive B-type lymphocytes were visualized within the small vessels and capillaries throughout	8 cycles of induction chemotherapy with methotrexate and rituximab and then 12 cycles of monthly maintenance methotrexate	The patient has survived for 23 months after the initial presentation and is still alive

Laboratory examinations revealed anemia and thrombocytopenia in three of the cases. An elevated serum lactate dehydrogenase (LDH) level was noted in all five patients. Cerebrospinal fluid (CSF) analyses were available for four patients, and only one patient’s CSF showed slight pleocytosis, while two patients’ CSF had elevated protein levels. Bone marrow biopsy was performed on three patients, and only one showed a population of small clonal B-cells, but without clinical significance (Table 1).

Neuroimaging results

**Figure 2:**
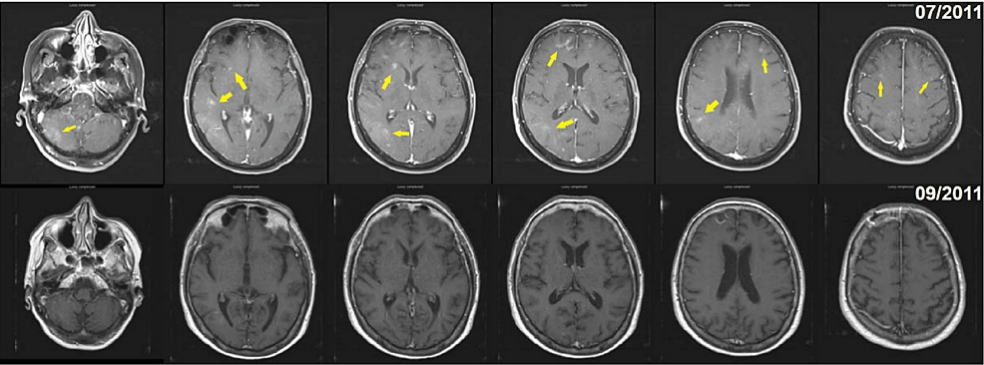
Resolved gadolinium-enhanced lesion after chemotherapy (case 5). (A) Prior to therapy (yellow arrow); (B) three months after the induction therapy.

All five patients underwent brain MRI imaging, with three patients receiving serial imaging during their clinical course. Neuroimaging findings were highly variable and nonspecific, including white matter lesions, infarcts, hemorrhages, or combinations of these. Non-specific white matter lesions were observed in all five patients. Infarct-like lesions were observed in three of five patients, manifesting as multiple hyperintense spots on T2/FLAIR signal and diffusion restriction scattered throughout bilateral cerebral hemispheres. Serial MRI brain imaging of two untreated patients showed recurrent dynamic progression, with both hemorrhagic (Figure 1A) and infarct lesions (Figure 1B), as well as diffuse multi-focal cortical/subcortical confluent T2/FLAIR hyperintensities (Figure 1C). In one patient who received CNS chemotherapy, the enhanced lesion completely resolved after the induction phase of chemotherapy, and serial surveillance MRI brain did not reveal any evidence of tumor relapse (Figures 2A, 2B). Detailed MRI findings are presented in Table 1 and Figures 1, 2. 

Pathological findings

**Figure 3:**
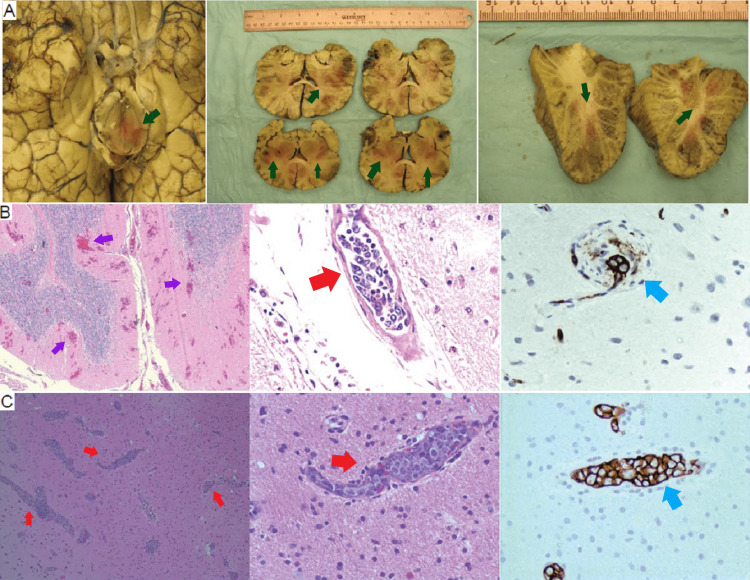
Pathological findings of IVLBL (case 1). (A) Gross anatomy of autopsied brain showed scattered parenchymal hemorrhages (dark green arrow) present in bilateral cerebral hemisphere, brain stem and cerebellum; (B) multifocal subtotal necrosis with small patches of incomplete neuronal loss, reactive astrocytes, and microhemorrhage (purple arrow); (B, C) H-E staining showed that cerebral and leptomeningeal vessels were plugged by numerous neoplastic large lymphoid cells, which were noncohesive and free in the lumina, and confined to the vessels (red arrow). Lymphoma cells in all cases were confirmed as B-cell lineage by immunohistochemistry staining using anti-CD20 (blue arrow).

The autopsy was performed on three patients who died within three months of clinical presentation. Gross pathological examination revealed multifocal parenchymal hemorrhagic or ischemic infarcts (Figure 3A). Patients with rapidly deteriorating neurological symptoms had parenchymal necrosis and microhemorrhages, while the patient with a relatively slow clinical course did not show necrosis or microhemorrhage (Figures 3B, C). Microscopic examination showed neoplastic large lymphoid cells plugging cerebral and leptomeningeal vessels (Figures 3B, C). The lymphoma cells were noncohesive and free in the lumina or confined in the vessels in autopsy or biopsy specimens from the brain or muscle. Immunohistochemistry staining confirmed B cell lineage of the lymphoma cells using anti-CD20 and/or anti-CD79a antibodies (Figures 3B, 3C, Table 1).

In the patient with multi-organ failure, concomitant extravascular infiltrates of neoplastic lymphocytes were observed, including diffuse infiltration to the spleen, liver, and kidney.

Clinical course, treatment, and survival analysis

Three patients with a rapid clinical course were given IV (intravenous infusion) steroid treatment for suspected CNS vasculitis, resulting in transient improvement of symptoms such as mental status, language difficulty, and seizures. However, the patients ultimately experienced a decline and died within three to four months of initial presentation.

In a patient with IVLBL affecting the lower extremity muscles, chemotherapy with the CHOP regimen (cyclophosphamide, hydroxydaunorubicin, Oncovin, and prednisone) excluding vincristine for six cycles was effective in improving symptoms of lower extremity weakness, generalized fatigue, and B-symptoms. Relapsing symptoms recurred four months after the diagnosis, and the patient was treated with two courses of a MIME (methyl-GAG, ifosfamide, methotrexate, etoposide) regimen and three doses of rituximab, resulting in symptom resolution, improved functional status, and survival for 13 months after initial presentation.

Another patient with CNS IVLBL began treatment with high-dose systemic methotrexate and rituximab as induction chemotherapy for eight cycles, resulting in dramatic improvement of symptoms of memory loss, aphasia, and seizures after the third cycle. Repeat MRI brain imaging showed resolving enhanced lesions (Figure 2). After induction, the patient continued to be maintained with systemic methotrexate therapy and had mild ataxia symptoms but was clinically stable with a stable brain MRI appearance on follow-up serial surveillance imaging. Patients who did not receive chemotherapy had a survival rate of three to four months, whereas the two patients undergoing chemotherapy survived for more than 12 months after the initial clinical presentation.

## Discussion

Our patients exhibited clinicopathologic characteristics consistent with previous reports, including a variety of common and diverse symptoms attributable to CNS involvement in both European and Asian IVLBL variants. All five patients experienced acute/subacute mental status changes, with three showing language deficits such as dysarthria and aphasia, two experiencing seizures, two presenting with ataxia, and two showing rapid progressive dementia. Two patients with focal sensory or motor deficits were confirmed to have cerebrovascular events. In addition to CNS symptoms, tumor cells can also involve other systemic organs and lead to various systemic symptoms, such as fever of unknown origin, general fatigue, hemophagocytic syndrome, anemia, and cutaneous involvement. For instance, one patient who presented with fulminant multi-organ failure showed infiltration of lymphoma cells into different organ vascular systems. Given the heterogeneity of these symptoms, identifying this disease in patients can be extremely challenging. When reviewing the clinical data for all five patients, a wide range of differential diagnoses and thorough work-up was carried out, and the initial working diagnosis varied in each individual.

Laboratory findings in IVLBL are not specific but can raise suspicion of the disease. Anemia is the most common abnormal finding, present in almost 65% of patients. Increased levels of LDH and b2-microglobulin were observed in more than 80%-90% of patients and are recommended to be included in the staging workup for IVLBL [1,2,15]. In patients with CNS involvement, radiological findings may include ischemia/infarction, intraparenchymal or subarachnoid hemorrhage, nonspecific white matter lesions, meningeal enhancement, and less commonly, mass lesions [18]. While MRI brain imaging findings can be interpreted logically in conjunction with pathological results, their diverse characteristics can be mistaken for CNS infection, inflammation, or CNS vasculitis. Attempts have been made to associate the dynamic evolution of CNS lesions with the natural course of untreated IVLBL cases, and it has been reported that the resolution of diffusion, FLAIR, or enhancing lesions is associated with clinical neurological improvement in response to chemotherapy, as in one of our patients who received chemotherapy [18]. However, non-specific imaging results have a limited contribution to the clinical diagnosis, and their correlation with the clinical picture is poor. Therefore, imaging data seldom serve as a predictive value for the outcome. The variable neurological manifestations are likely pathophysiologically secondary to the intravascular locations of the malignancy, causing intravascular tumor thrombi and multifocal cerebral infarcts. associated with other systemic presentations of a non-Hodgkin's lymphoma.

Accurate diagnosis of IVLBL requires tissue biopsies. Tumor involvement can occur in any organ, and a cutaneous lesion can be biopsied if there is high clinical suspicion [1]. In certain clinical circumstances, a muscle or muscle/nerve biopsy may provide a safe and convenient location for biopsy. In one of our patients, EMG showed severe inflammatory myopathy, which primed the muscle biopsy. Visceral organ biopsy under CT guidance has been reported, but severe complications, such as intraperitoneal hemorrhage, can occur, as in one patient who died of multi-organ failure [15]. When patients present with CNS symptoms as their initial clinical presentation, early brain biopsy is crucial for an accurate diagnosis and is obligatory to initiate chemotherapy. However, most patients are often diagnosed postmortem due to rapid deterioration and a very short clinical course.

While IVLBL is sensitive to systemic chemotherapy due to the lymphoma cells being confined within the lumina of blood vessels, there are no randomized controlled trials comparing treatment options and clinical outcomes. DiGiuseppe and Ferreri reported that anthracycline-containing chemotherapies improved clinical outcomes with a response rate of nearly 60% and a three-year overall survival rate of 33% [7,19]. Recently, rituximab, a chimeric monoclonal antibody against CD20, has produced a profoundly positive response in cases of IVLBL as both initial and salvage therapy. Retrospective studies from Asian and European groups have suggested that the introduction of rituximab improved the outcome of IVLBL, with both response rate (>80%) and survival duration being superior to those in the pre-rituximab era [11]. However, the sites of disease play an important role in therapeutic efficacy, and CNS involvement was reported as the principal site of treatment failure. No difference in CNS relapse rate was seen between patients treated with chemotherapies with or without rituximab, indicating that CNS relapse in IVLBL could not be overcome by the introduction of rituximab [20]. Therefore, a more intensive combination and CNS-oriented therapy are needed in patients with CNS involvement. Reagents with higher bioavailability in the CNS, such as methotrexate and cytarabine, should be considered [16]. From our observation, the patient who received methotrexate/rituximab induction and methotrexate maintenance therapy tolerated the regimen well and achieved nearly two-year survival after the initial clinical presentation. However, more studies are needed to evaluate the efficacy of different treatment options, especially for CNS involvement in IVLBL.

## Conclusions

IVLBL is commonly known as a “great masquerader” due to its tendency to present with a variety of symptoms. The prognosis of IVLBL depends on several factors, including early and accurate pathological diagnosis, prompt chemotherapy with rituximab, and the cutaneous variant of the disease. Our observation suggests that patients with peripheral involvement such as skin or muscle lesions, without visceral organ or CNS lesions, or those with CNS lesions lacking pathological evidence of tissue necrosis, reactive astrogliosis, or microhemorrhage may have a better treatment outcome. Although the pathologic features of IVLBL are thought to be associated with vascular occlusion, the underlying mechanisms of neoplastic lymphoma cells' intravascular localization and predilection for the CNS are still unclear. Further research is needed to elucidate the disease's pathophysiology and develop more effective treatments.

IVLBL is an aggressive and rare subtype of extranodal large B-cell lymphoma with unique pathological characteristics. Its clinical presentation is highly diverse and can mimic several other pathogenic entities, making early diagnosis challenging. Non-specific imaging findings, a broad range of clinical symptoms, and a rapid clinical course can further complicate early diagnosis. The patient's best chance for survival is contingent upon a timely and accurate pathological diagnosis followed by aggressive chemotherapy.
